# Extracellular lactate cooperates with limited glucose and glutamine to sustain breast cancer cell survival by providing ATP, NADPH, amino acids, and glutathione

**DOI:** 10.1186/2049-3002-2-S1-P49

**Published:** 2014-05-28

**Authors:** Jennifer Molina, Pietro Morlacchi, Leslie Silva, Jennifer Dennison, Gordon Mills

**Affiliations:** 1 Systems Biology, UT MD Anderson Cancer Center, Houston, TX, USA; 2 Genomic Medicine, UT MD Anderson Cancer Center, Houston, TX, USA; 3 Bioinformatics & Computational Biology, UT MD Anderson Cancer Center, Houston, TX, USA

## Background

Cancer progression occurs upon mutations on regulatory genes that control biological functions including cellular bioenergetics. Such perturbations lead to a metabolic switch that favors aerobic glycolysis and lactate production over oxidative phosphorylation. This process is known as the Warburg effect and results in a lactate-rich tumor microenvironment. The apparently wasteful mechanism has raised the question of why cancer cells switch from the high ATP producing TCA cycle/OXPHOS to glycolysis; and whether lactate serves a biological function in energy metabolism since a high lactate environment correlates with worse patient prognosis in several malignancies including breast cancer. Our goals were to first, determine the fate of carbons from lactate in breast cancer cells; and second, determine the mechanism of lactate metabolism.

## Materials and methods

In the current study we used several assays including: survival assay under nutrient stress conditions, NMR spectroscopy, mass spectrometry, mitochondrial respiratory analysis by FX analyzer, nutrient quantification YSI analysis, enzyme inhibition by siRNA and small molecule inhibitors.

## Results

Using breast cancer cells as a model system, we found that extracellular lactate promotes a significant effect on cell survival under nutrient stress conditions where glucose or glutamine is limited. Exogenous lactate was taken up by cells, and significantly increased NADH levels and oxygen consumption for ATP production. NMR flux analysis with [U-13C]-L-lactate revealed that lactate was metabolized for production of key non-essential amino acids including: glutamine, glutamate, alanine, aspartate, glycine, and cysteine. Our data also showed that lactate metabolism increased ROS production, due to a switch toward oxidative phosphorylation, which signaled cells to produce NADPH and initiate glutathione production from the lactate-derived amino acids glutamate, glycine, and cysteine. Mass spectrometry flux analysis demonstrated that a significant percentage of glutathione carbons were derived from lactate. Additionally, inhibition of glutathione synthesis by BSO treatment abrogated the survival effect of lactate and was rescued by treatment with glutathione. Furthermore, lactate-derived glutamine was re-metabolized into glutamate via GLS and to α-ketoglutarate via GLUD for NADPH production.

## Conclusions

Our data show that breast cancer cells uptake lactate under nutrient stress conditions and metabolize it to maintain energy homeostasis (ATP), fuel the TCA cycle, and for *de novo* synthesis of non-essential amino acids that play a major role toward redox balance by synthesis of glutathione. In addition, the limited glucose and glutamine cooperated with lactate to promote the metabolic adaptation. Our data suggest that lactate may promote a pathway to circumvent metabolic targeted therapy.

